# An Overview of Metabolic Activity, Beneficial and Pathogenic Aspects of *Burkholderia* Spp.

**DOI:** 10.3390/metabo11050321

**Published:** 2021-05-17

**Authors:** Hazem S. Elshafie, Ippolito Camele

**Affiliations:** School of Agricultural, Forestry, Food and Environmental Sciences, University of Basilicata, Via dell’Ateneo Lucano 10, 85100 Potenza, Italy; ippolito.camele@unibas.it

**Keywords:** secondary metabolites, plant diseases, human and animal pathogens, plant growth promoting, biological control

## Abstract

*Burkholderia* is an important bacterial species which has different beneficial effects, such as promoting the plant growth, including rhizosphere competence for the secretion of allelochemicals, production of antibiotics, and siderophores. In addition, most of *Burkholderia* species have demonstrated promising biocontrol action against different phytopathogens for diverse crops. In particular, *Burkholderia* demonstrates significant biotechnological potential as a source of novel antibiotics and bioactive secondary metabolites. The current review is concerned with *Burkholderia* spp. covering the following aspects: discovering, classification, distribution, plant growth promoting effect, and antimicrobial activity of different species of *Burkholderia*, shedding light on the most important secondary metabolites, their pathogenic effects, and biochemical characterization of some important species of *Burkholderia,* such as *B. cepacia*, *B. andropogonis*, *B. plantarii*, *B. rhizoxinica*, *B. glumae*, *B. caryophylli* and *B. gladioli*.

## 1. Genus Burkholderia

### 1.1. Discovering, Classification and Distribution

In 1942, Burkholder described one of the first *Burkholderia* species, *Phytomonas caryophylli* [1], later known as *Pseudomonas caryophylli*. In 1949, Burkholder also described another bacterium that caused rot in onion bulbs, as reported by vegetable growers in New York State in the mid-1940s, and gave it the species name ‘cepacia’, which was later known as *Pseudomonas cepacia* [2]. *Burkholderia* spp. was included for many years in the genus *Pseudomonas* due to broad and vague phenotypic characteristics [3]. However, rRNA–DNA hybridization analyses during the early 1970s indicated considerable genetic diversity among members of this genus which was divided into five rRNA homology groups [4]. Subsequent genotypic analyses have confirmed that these five groups are only distantly related to each other. Consequently, *Pseudomonas* was restricted to homology group I containing the type species *Pseudomonas aeruginosa* [5]. In 1992, the seven species belonging to rRNA homology group II (*Pseudomonas solanacearum*, *P. pickettii*, *P. cepacia*, *P. gladioli*, *P. mallei*, *P.* *pseudomallei* and *P. caryophylli*) were transferred to the novel genus *Burkholderia* [6]. The members of genus *Burkholderia* have a broad distribution occurring commonly in soil, water, in symbiosis with plants and fungi and in association with animals and humans [7,8]. *Burkholderia* are motile and gram negative (G-ve) rods that may be straight or slightly curved. They are aerobic, catalase positive, urease positive, non-spore formers and non-lactose fermenting [9].

PCR can be used to distinguish between the different *Burkholderia* species. The ribosomal RNA gene is highly conserved and universally distributed in all living things, and therefore the difference in the DNA sequences between 16S and 23S rRNA genes can be used to differentiate between different species [10].

### 1.2. Species of Burkholderia

**Figure 1:**
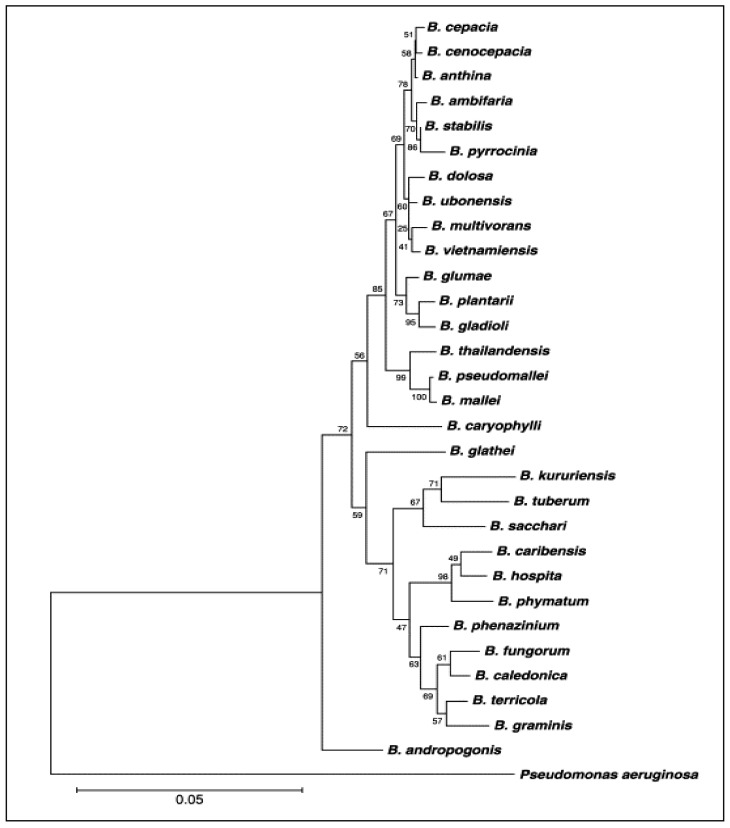
Phylogenetic tree of genus *Burkholderia* based on 16S rRNA gene sequence. This phylogenetic tree is in agree with Coenye and Vandamme [12].

The genus Burkholderia contains about 35 validly named species (Table 1): *B. alpina*, *B. ambifaria*, *B. andropogonis*, *B. anthina*, *B. arboris*, *B. caryophylli*, *B. cenocepacia*, *B. cepacia*, *B. contaminans*, *B. diffusa*, *B. dolosa*, *B. gladioli*, *B. glumae*, *B. humptydooensis*, *B. lata*, *B. latens*, *B. mallei*, *B. metallica*, *B. multivorans*, *B. oklahomensis*, *B. plantarii*, *B. pseudomallei*, *B. pseudomultivorans*, *B. puraquae*, *B. pyrrocinia*, *B. rhizoxinica*, *B. seminalis*, *B. singaporensis*, *B. singularis*, *B. stabilis*, *B. stagnalis*, *B. territorii*, *B. thailandensis*, *B. ubonensis* and *B. vietnamiensis *[11]. Figure 1 presents the phylogenetic tree based on the gene sequence of 16S rRNA, showing the position species included in the genus Burkholderia as reported by Coenye and Vandamme [12].

## 2. Plant Growth Promoting Effect of *Burkholderia* Spp.

*Burkholderia* spp. involve diverse mechanisms of action for promoting the plant growth, including rhizosphere competence for secretion of allelochemicals, production of antibiotics and siderophores such as iron chelating compounds [13,14,15]. Ornibactins is considered the predominant siderophores produced by *Burkholderia* spp. [16].

Pandey et al. [17] reported that the plant growth promoting activity of MSSP strain of genus *Burkholderia* was determined by some factors such as: 1-aminocyclopropane-1-carboxylic acid deaminase production [18], nitrogen fixation, phosphate solubilization, production of indol acetic acid (IAA), siderophores, and hydrogen cyanide (HCN) [17]. In addition, the same strain showed also an antagonistic activity against different phytopathogens [19].

Karakurt and Aslantas [20] reported that the application of *B. gladioli* increased the annual shoot diameter of some apple cultivars. *Burkholderia* spp. strain PsJN is considered an effective plant growth-promoting bacterium since it promotes the growth of potatoes, vegetables and grapevines by producing a high level of 1-aminocyclopropane-1-carboxylic acid deaminase which able to reduce the level of inhibitory hormone ethylene [21]. In addition, Compant et al. [15] studied the growth promoting effect of *Burkholderia* sp. strain PsJN on *Vitis vinifera* and concluded that all inoculated plants with this strain have performed better than those non-bacterized and the relative fresh weights of roots and aerial parts were significantly increased compared to the non-bacterized plants.

Karakurt and Aslantas [20] evaluated the effects of some different strains of *B. gladioli* on the growth and the leaf nutrient content of Starking Delicious, Granny Smith, Starkrimson Delicious, Starkspur Golden Delicious and Golden Delicious apple cultivars grafted on semi-dwarf rootstock and observed an increase of leaf number and area as well as the number of annual shoots and their diameters. Furthermore, the latter authors also reported that the application of *B. gladioli* has increased the manganese content and did not affect the concentration of sodium and calcium in the leaves [20].

## 3. Use of *Burkholderia* Spp. as Biocontrol Agent

Most of *Burkholderia* species can be used potentially as biocontrol agents against phytopathogenic fungi, bacteria, protozoa and nematodes in many different crops such as: corn, sweet corn, cotton, grapevine, pea, tomato and pepper [22,23]. On the other hand, some *Burkholderia* species were commercialized and effectively used as biocontrol agents in agriculture [24]. Recently, many researchers have conducted different studies for evaluating the antagonistic effect of *Burkholderia* spp. for controlling plant diseases since these bacteria are known as producer of many bioactive metabolites such as bacteriocins, alkaloids, lipopeptides and polypeptide [25].

In particular, Holmes et al. [26] studied the capacity of *B. cepacia* in degradation of chlorinated aromatic substrates in certain synthetic pesticides. Some other strains of *Burkholderia* produce enzymes able to degrade non-nutritive substrates, such as trichloroethylene (TCE), a major ground water contaminant used in the dry cleaning industry and in degreasing solvents [27]. Other species such as *B. bryophila* and *B. megapolitana* showed antifungal activity against some phytopathogens as well as plant growth-promoting properties [28]. Another study has been conducted by Barka et al. [21] reported that strain PsJN of *Burkholderia* has showed a biocontrol effect against *Botrytis cinerea* and proved also its growth promoting effect on the grapevine [21].

In addition, several strains of *B. gladioli* showed an effective *in vitro* antagonistic activity against a wide range of fungal and bacterial species [29,30,31]. The above-mentioned species could completely inhibit the conidial germination of *Penicillium digitatum* and *Botrytis cinerea,* as reported by Walker et al. [32]. In addition, the metabolites produced by *B. gladioli* caused a significant inhibition of *Penicillium expansum,* as reported by Elshafie et al. [29]. Apparently, a growth suppression of some pathogenic fungi by *B. gladioli* strains was more efficient when the bacterial cultures were used than the culture filtrates and this verified the synergic effect of several bioactive substances [29]. However, the antagonizing activity of *B. gladioli* explained by the combination between competition for nutrients, space and production of antifungal metabolites [30].

Several recent studies showed antimicrobial activity of *B. gladioli* pv. *agaricicola* against some serious phytopathogens [29,30,31,32,33,34]. In particular, the pathovar *agaricicola* showed antagonizing activity against wide range of important phytopathogenic fungi, including *Botrytis cinerea*, *Aspergillus flavus*, *Aspergillus niger*, *Penicillium digitatum*, *Penicillium expansum*, *Sclerotinia sclerotiorum* and *Phytophthora cactorum* [29]. In the same context, Elshafie et al. [30] reported that four studied strains of *B.* *gladioli* pv. *agaricicola* (ICMP: 11096, 11097, 12220 and 12322) have exerted antifungal activity against above mentioned phytopathogenic fungi by producing diffusible metabolites and extracellular hydrolytic enzymes. The same authors have attributed this bioactivity to the production of two bioactive fatty acids identified as methyl stearate and ethanol 2-butoxy phosphate with mass spectrum m/e 298 and 398, respectively [30].

Another recent study reported that the application of *B. gladioli* pv. *agaricicola* strain ICMP 12322 was able to enhance the disease protection and improve the consistency of biological control against tomato-wilt disease caused by *Verticillium dahliae* [34].

## 4. Induction of Plant Systemic Resistance (ISR)

The microbial community in soil can play a vital role in stimulation the plant growth and also can suppress the deleterious effect of other soil microorganisms [35]. In particular, *Rhizobacteria* can reduce the activity of pathogenic microorganisms not only through the microbial antagonism, but also by inducing the plant to defend itself. This phenomenon, named “induced systemic resistance” (ISR), was first described by Van Peer et al. [36]. ISR can be triggered by some specific strains of plant growth promoting bacteria (PGPB) through the production of some plant signaling molecules [37].

Some endophytic bacterial strains belonging to the genus *Burkholderia* and *Bacillus* are considered effective biological control agents [38]. The beneficial effects of *Burkholderia* spp. in agricultural could be explained by induction of plant resistance against abiotic stresses through ISR and others mechanisms [23]. *B. phytofirmans* strain PsJN-grapevine interaction, a host defense reaction coinciding with phenolic compounds accumulation and strengthening of cell walls in the exodermis and in several cortical cell layers [39]. Sharma and Nowak [40] and Bordiec et al. [41] reported the biocontrol effect of strain PsJN against *Verticillium dahliae* and *Botrytis cinerea*, the causal agents of wilt disease and grey mould, respectively.

## 5. *Burkholderia*’ Diseases on Human and Animals

Several species of *Burkholderia* have been reported as rich of virulence factors such as: presence of a flagella, reactive oxygen species resistance and resistance to several antimicrobial drugs [42]. These above mentioned traits of *Burkholderia* enable them to be adapted perfectly in their different ecological niches [42]. Many species of *Burkholderia* are known as phytopathogens [43,44] however there are other species belong to *Burkholderia* have demonstrated some opportunistic infection to animal and human. In particular, *B. pseudomallei* and *B. mallei* were considered as pathogens for animals and humans and they are both resistant to a number of antibiotics [45]. *B. mallei* is responsible for glanders disease, which mostly affected animals, such as horses, mules, donkeys and rarely humans [46]. Whereas, *B. pseudomallei* is the causal agent of melioidosis, the disease in tropical countries [47]. On the other hand, *B. cepacia* complex (Bcc) has a natural occurrence in the environment and has both beneficial and detrimental effects on plants, however it is considered an opportunistic human pathogen. Bcc causes severe lung infections in cystic fibrosis patients and it is often resistant to common antibiotics and able to degrade natural and man-made pollutants [48,49,50].

## 6. Microbial Secondary Metabolites

Most of living organisms, such as invertebrates, plants and microorganisms, are lacking the immune system, hence they have developed the capacity to produce bioactive secondary metabolites including some toxic substances against other harmful microorganisms. These natural products act as specific defense systems against other organisms [51,52]. Secondary metabolites are compounds that are not required for the growth or reproduction but play a vital role in inhibiting the growth of harmful organisms with which they compete and can also inhibit their biologically important processes [53].

Microbial secondary metabolites (MSM) are the most promising source of novel natural products; hence their discovery and characterization are the objective of many researches for controlling important phyto- and human pathogens [53,54,55].

In addition, MSM are low-molecular-mass products of secondary metabolism, usually produced during the late growth phase of microorganisms and their production arises from intracellular intermediates (amino acids, sugars, fatty acids, etc.). MSM are very important for the human health and economics of our society [53,54].

There are thousands of important known MSM, among them penicillin, which was discovered in 1940, obtained from *Penicillium* moulds, such as *P. chrysogenum* and *P. rubens*, which began the era of antibiotics. The penicillin has been recognized as one of the greatest advances in therapeutic medicine [56].

*Aspergillus terreus* has been reported to produce biological drugs known as statins. The statins are class of drugs that inhibit HMG-CoA reductase and lead to lower cholesterol level [57]. On the other hand, a new substance named lovastatin, with a similar structure of statin, has been extracted from *Monascus purpureus* and *Monascus ruber* [58].

### 6.1. Secondary Metabolites Produced by Genus Burkholderia

Bacterial secondary metabolites (BSM) are considered one of the most promising sources among the novel bioactive pharmaceutical compounds. In particular, *Actinobacteria* are considered the major source of bioactive BSM, such as different antibiotics, which usually used for human being and animals [59]. Generally, the majority of discovered antimicrobials substances have been isolated from *Actinomycetes* especially from genus *Streptomyces* Waksman and Henrici. Among the most important common antibiotics: tetracycline and aminoglycoside or glycopeptide [60].

Several *Burkholderia* species, considered as beneficial bacteria in the natural environment, have the ability to produce compounds with antimicrobial activity [61] and can be used as biocontrol agents for phytopathogenic fungi and able to inhibit the growth of other bacteria, protozoa and nematodes in many different crops, such as corn, sweet corn, cotton, grapevine, pea, tomato, and pepper [22]. In general, *Burkholderia* demonstrate significant biotechnological potential as a source of novel antibiotics and bioactive secondary metabolites [62,63].

In particular, genus *Burkholderia* showed high ability to produce several extracellular hydrolytic enzymes such as chitinase, protease, cellulase, amylase and glucanase [64], which may have important applications in both pharmaceutical industry [29,65]. On the other hand, genus *Burkholderia* produced also a wide range of secondary metabolites such as pyrrolnitrin, phenazine, cepabactin, and other bioactive diffusible and volatile compounds [34,66,67,68,69].

In the current review, the most important species of genus *Burkholderia* were reported as following: *B. cepacia* Palleroni and Holmes (Yabuuchi et al.), *B. andropogonis* Smith (Gillis et al.), *B. plantarii* Azegami et al. (Urakami et al.), *B. rhizoxinica* Partida-Martinez et al., *B. glumae* Kurita and Tabei (Urakami et al.), *B. caryophylli* Burkholder (Yabuuchi et al.), and *B. gladioli* Severini (Yabuuchi et al.).

#### 6.1.1. *Burkholderia cepacia*

**Figure 2:**
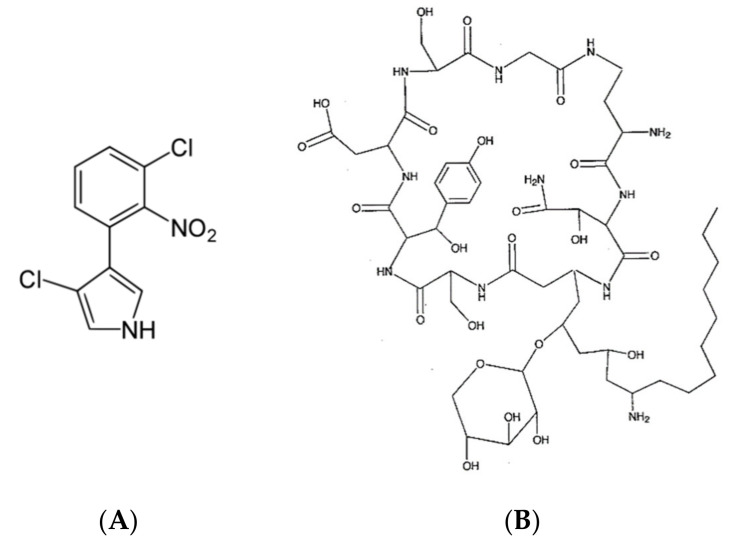
Secondary metabolites produced by *Burkholderia cepacia.* Where (**A**) 3-chloro-4-(2′nitro-3′cloro-phenyl) pyrrole pyrolnitrin and (**B**) Cepacidine A.

*B. cepacia* produces a bioactive compound called 3-chloro-4- (2′nitro-3′cloro-phenyl) pyrrole pyrrolnitrin [25,70] (Figure 2A) which showed antimicrobial activity against some pathogenic fungi, yeast and Gram-positive (G+ve) bacteria as reported by Arima et al. [71,72]. Arima et al. [71] reported the molecular formula of pyrrolnitrin C_10_H_6_O_2_N_2_Cl_2_ and observed that this compound is a pale-yellow crystal and can loss its bioactivity if exposed to sun light or acidic conditions. Pyrrolnitrin is well solubilized in different organic solvents such as methanol, ethanol, butanol, acetone, ethyl acetate, etc., whereas it is slightly solubilized in water [71]. Rahman et al. [70] reported that the antifungal activity of *B. cepacia* is due to the chemical toxicity nature of pyrrolnitrin, which can penetrate the cell membrane and leads to the protoplasmic dissolution and disintegration and finally inhibit the cell growth. On the other hand, the same authors explained that the vacuolar appearance of the mycelium may be due to the antibiotic metabolites [70].

Another important antifungal compound produced by *B. cepacia* is called cepacidine A (Figure 2B) [73] which demonstrated a strong activity against *Trichophyton* spp. and *Epidermophyton* spp. Cepacidine A consists of two closely related compounds, cepacidin A1 and A2, with molecular weights of 270.29 Da and 286.29 Da, respectively, in a ratio 9:1. Furthermore, Parker et al. [74] discovered cepacina A and B, two important bioactive compounds, produced by *B. cepacia* which were able to inhibit the growth of *Staphylococcus* spp. and some G-ve bacteria. In particular, cepacina B is significantly more active than cepacina A against both G+ve and G-ve [74], whereas both compounds are slightly active against streptococci bacteria. On the other hand, Santos-Villalobos et al. [75] found that siderophores, volatile metabolites produced by *B. cepacia*, were able to control the growth of *Colletotrichum gloeosporioides*.

#### 6.1.2. *Burkholderia andropogonis*

*B. andropogonis* was first described by Smith [76] as the causal agent of stripe disease of sorghum [77]. It was named before as *Pseudomonas andropogonis* and then transferred to genus *Burkholderia* by Gillis et al. [78]. *B. andropogonis* has been reported also to cause bacterial stripe disease sudangrass, teosinte, johnsongrass, field corn, broomcorn, and sweet corn [79].

**Figure 3:**
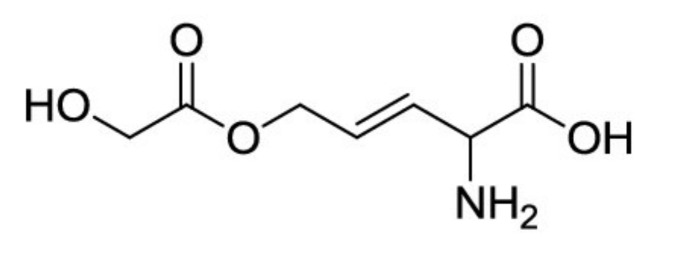
Chemical structure of rhizobitoxine.

This bacterium produces amino enol ether rhizobitoxine which is responsible for the chlorosis of soybean [80]. The structure of rhizobitoxine, illustrated in Figure 3, was identified by Owens et al. [81]. Rhizobitoxine, with a molecular weight of 190 Da, was able to inhibit the ethylene biosynthesis in apple tissues [82] and reduce the defense reaction by the host plants [83]. The capacity of rhizobitoxine to inhibit the ethylene production may enhance the nodulation and competitiveness in *Macroptilium atropurpureum* and *Vigna radiata* [84]. Yasuta et al. [85] reported that rhizobitoxine has significantly inhibited 1-aminocyclopropane-1-carboxylate synthase bLE-ACS2 from tomato, which considered the key enzyme in the pathway of ethylene biosynthesis.

Furthermore, Sugawara et al. [83] explained that rhizobitoxine has strongly inhibited the enzyme [1-aminocyclopropane-1-carboxylate (ACC) synthase] in the ethylene biosynthesis pathway which would explain the early observation of rhizobitoxine inhibition of ethylene evolution in apple tissues. On the other hand, Sugawara et al. [83] reported also the positive role of rhizobitoxine in the symbiosis between *Bradyrhizobium elkanii* strains and their host legumes. The latter coauthors also reported that rhizobitoxine, as an analog of cystathionine, can irreversibly inhibit β-cystathionase in bacteria and plants.

#### 6.1.3. *Burkholderia plantarii*

The name of this species derived from the Latin word *plantarium* (seedbed). *B. plantarii* is responsible for root rot, seedling blight, chlorosis and reduction of root growth of rice [86]. *B. plantarii* was found to be distributed on the weeds in fields and in seed stored at room temperature and it was often isolated in association with *B. glumae* indicating that these two species may have similar transmission path and life cycle [86].

*B. plantarii* produces a compound called tropolone with molecular weight of 122 Da, which has phenolic and acidic characteristics with antimicrobial activity and phytotoxic effect on rice. Tropolone, identified in 1945, is a non-benzenoid aromatic compound and has similar characteristics of phenols and acids [86]. Trust [87] reported that tropolone showed bacteriostatic and bactericidal effect against wide range of bacterial species such as *Bacillus subtilis*, *Escherichia coli*, *Pseudomonas aeruginosa*, *Salmonella typhi* and *Serratia marcescens*. The mechanism of the biological activity of this compound is based mainly on its ability to penetrate the plasma membrane and cell wall of microbes and increasing the cell permeability and leads to cell lysis and subsequent loss of cell contents after rupture of the bleb [87]. Azegami et al. [86] observed that the mere addition of iron to the MA broth culture media has greatly enhanced the growth of *P. plantarii*. However, the mere addition of ferric chloride has markedly reduced the amount of dissolved tropolone [86].

Furthermore, this bacterium produces another two bioactive compounds identified as: 2-methylene-3-imino-5-L (carboxy-L-valine)-pyrrolidine and 2-methylene-3-imino-5-L (carboxy -L-treoninil)-pyrrolidine with molecular weights of 242.11 Da and 240.13 Da, respectively [88]. Mitchell and Katrina [88] reported that the last two compounds are amino acid conjugates to a new iminopyrrolidine carboxylic acid structure and this is in keeping with the amino acid conjugation characteristic related to many natural compounds that exhibited biological activity [88]. The last two bioactive compounds are able to inhibit *Erwinia aylovora*, which is responsible for fire blight disease of pome fruit, especially for apple and pear trees [88]. Moreover, *B. plantarii* strain DSM 9509 produces extracellular rhamnolipids when grown in glucose supplemented rich medium [89]. Rhamnolipids have been used in different applications as detergents and in the pharmaceutical industry [89].

#### 6.1.4. *Burkholderia rhizoxinica*

**Figure 4:**
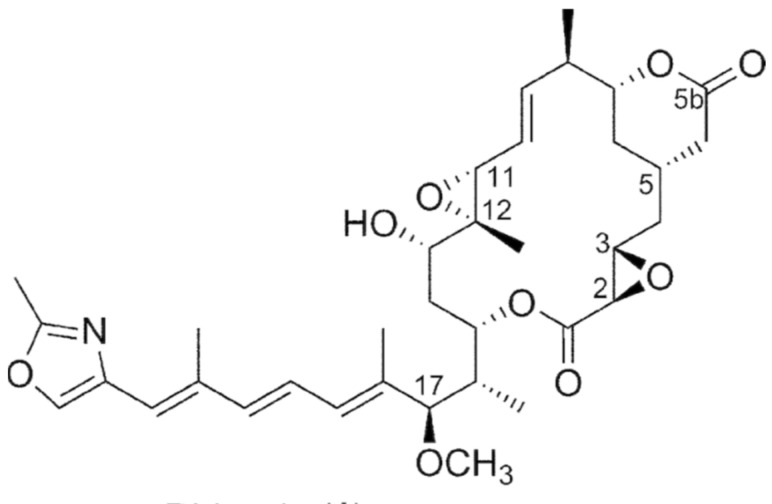
Chemical structure of rhizoxin.

The specific name of this species refers to its ability to produce the rhizoxin antibiotic (Figure 4) [90,91]. *B. rhizoxinica* is able to grow under aerobic and microaerophilic conditions, but not in an anaerobic atmosphere containing CO_2_ [91]. Recently, it has been reclassified as *Paraburkholderia rhizoxinica* Partida-Martinez (Sawana) [92]. *B. rhizoxinica* is an intracellular symbiont endophytic and was isolated from the phytopathogenic fungus *Rhizopus microsporus*, a common pathogen for food and feed stuff which causes rice seedling blight [93,94,95]. *B. rhizoxinica* is now associated with the ability of *Rhizopus* to cause rice seedling blight [96]. Rhizoxin is an important virulence factor for infection of plants and has phytotoxic, antifungal and anticancer activities [91,96,97].

Some clinical isolates of *B. rhizoxinica* might have the capacity to produce cytotoxic polyketides [98], which could aggravate the human infection due to its anti-mitotic activity in mammalian cells [91,94].

#### 6.1.5. *Burkholderia glumae*

*B. glumae*, the causal agent of bacterial grain rot and seedling rot of rice, was isolated from hot and high relative humid areas [99]. *B. glumae* was first reported in Japan, but later it was distributed in different countries producing rice such as: Japan, Thailand, Vietnam, South Korea, Malaysia, Philippines, Sri Lanka, United States, Panama, Nicaragua, Costa Rica, and Colombia [100]. The incidence of *B. glumae* has been increased recently due to climate changes, as well as the deficiency of appropriate management and biocontrol strategies [100].

**Figure 5:**
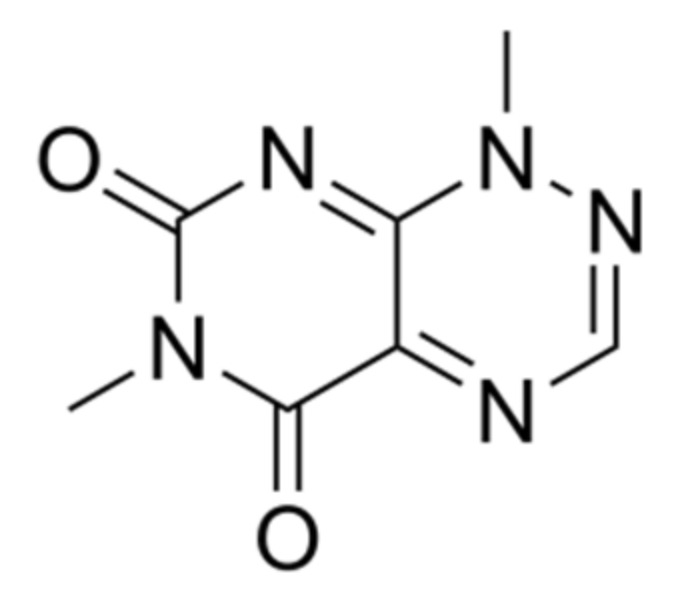
Chemical structure of toxoflavin.

*B. glumae* produces a range of secondary metabolites and lipase on agar media. In particular, antibiotic production is stimulated by some substrates presented in agar such as K+, Ca^2+^, Mg^2+^ and NH^4+^. Among the active metabolites produced by this bacterium is toxoflavin (Figure 5), which plays a role in pathogenicity of this bacterium and is involved in the rice grain rot [101].

Toxoflavin is a bright yellow color and is highly toxic to plants, fungi, animals and microorganisms [102]. In addition, the toxicity of toxoflavin to plants has led to severe losses in rice crops around the world [102]. The production of this molecule is influenced by temperature whereas the maximum suitable temperature is at 37 °C [103]. Lee et al. [102] reported also that the toxoflavin biosynthesis process is regulated by QS mechanism depending on the homoserine lactone synthesized by cognate receptors TofI and TofR, through the activation of ToxJ and ToxR as transcriptional regulators of toxoflavin biosynthesis.

#### 6.1.6. *Burkholderia caryophylli*

*B. caryophylli*, a parasitic endophyte infecting vascular plants, was previously classified as *Pseudomonas caryophylli* (Burkholder) Starr & Burkholder, is the causal agent of wilt stem cracking and a progressive rot of stems and roots of carnation [104]. It used to be a major problem in carnation production in the USA [105]. *B. caryophylli* is a soil borne bacterium that overwinters in the rhizosphere of soil forming close interactions with the host plant and soil itself. This species can survive in infected host debris and can infect many different species of the dianthus plant [106].

*B. caryophylli* produces caryoynencine toxin with molecular weight of 280.31 Da [104]. Caryoynencines are unstable C18 carboxylic acids with conjugated dienetetrayne and polymerize structures [107]. On the other hand, caryoynencine showed potent antimicrobial activity against G+ve and G-ve bacteria especially against the growth of methicillin-resistant *Staphylococcus aureus* (MESA) [107]. In addition, some analogs of caryoynencine exhibited a broad spectra of activity against the following pathogenic fungi: *Tricophyton mentagrophytes*, *T. interdigitale* and *T. rubrum* which are the causal agents of onychomycosis and tinea pedis in humans [107].

#### 6.1.7. *Burkholderia gladioli*

*B. gladioli* is an aerobic G-ve rod-shaped bacterium that may cause disease in human, plants and mushrooms [108]. This species is included in phylum *Proteobacteria*; class *Betaproteobacteria*; order *Burkholderiales*; family *Burkholderiaceae* and genus *Burkholderia*. *B. gladioli* can be distinguished from the other *Burkholderia* species because it is oxidase negative [108].

*B. gladioli* was initially identified in gladiolus and successively, associated with other plant diseases such as onions, iris, freesia, dendrobium, cymbidium, tulip, green gram and rice [109]. Disease symptoms varied from the spotting of foliar parts to scabbing and rotting of storage tissues [109]. In the last decade, different strains of *B. gladioli* have demonstrated the ability to infect human causing severe pulmonary infections in cystic fibrosis and other immune-compromised human patients [110,111]. *B. gladioli* is closely related to a member of *B. cepacia* complex that includes ten closely related species which are all plant pathogens [112].

*B. gladioli* is negative for indole production, nitrate utilization and lysine decorboxylation [9]. On the molecular level, two primers (GLA-f 5′-CGAGCTAATACCGCGAAA-3′ and GLA-r 5′-AGACTCGAGTCAACTGA-3′) were used for the amplification from 16S to 23S region in the *B. gladioli* genome [10]. The obtained amplicon by using these two above mentioned primers in PCR assay was approximately 300bp [10].

*B. gladioli* contains four pathovars. Three pathovars, *gladioli*, *alliicola* and *agaricicola* causing soft rots on gladiolus, onion bulbs and mushroom, respectively [7,29,113]. Whereas, the fourth pathovar, cocovenenans causes food spoilage which can be toxic to animal and human being consumers [114]. Differentiation of these four pathovars was made based on hosts, molecular basis and biochemical properties [7,113,114,115,116,117].

*B. gladioli* pv. *gladioli* Severini (Yabuuchi et al.) is the causal agent of soft rot of stem bases and corms [118]. On the fern *Asplenium nidus* (bird’s nest fern), leaf spot and blight have been observed, causing extensive losses in many nurseries in Florida, USA [119].

*B. gladioli* pv. *alliicola* Burkholder (Starr and Burkholder) has been isolated recently from onion in the Northeastern Slovenia infecting about 30% of onion bulbs. The internal layers were found to have water-soaked and brown-colored lesions [120]. This pathovar exhibited two different white-yellowish color colonies; one has a slightly wrinkled surface where the other has a smooth surface.

*B. gladioli* pv. *cocovenenas* van Damme et al. (Gillis et al.) was isolated from a petroleum- contaminated soil [121] and described as producer for lethal toxins (Bongkrekic acid and toxoflavin) which are toxic to animals [114,122] and was also reported sometimes to cause pneumonia for humans [114].

*B. gladioli* pv. *agaricicola* (*Bga*) Yabuuchi is considered an important pathogen for mushroom [123] because it may cause a significant crop loss [109]. *B. gladioli* pv. *agaricicola* causing soft rot and cavity disease on mushroom [114,124]. In particular, some strains of this pathovar causes soft rot on a number of commercially important mushrooms, such as *Lentinula edodes*, *Pleurotus ostreatus*, *Flammulina velutipes*, *Pholiota nameko*, *Hypsizygus marmoreus* and *Grifola frondosa* in Japan and different cultivated *Agaricus* species in New Zealand and Europe [109,124].

Based on the proposal of Yabuuchi [6] who proposed a new genus, *Burkholderia*, to include members of the “pseudomallei group”, the pathovar “*agaricicola*”, previously classified as *Pseudomonas gladioli* pv. *agaricicola* [125], was subsequently transferred to the new genus *B. gladioli* pv. *agaricicola* [114,126,127].

Secondary metabolites produced from *Bga* are implicated within the quorum sensing (QS) phenomenon [124,128]. This mechanism enable the bacterial cells to communicate to each other by responding to different signal molecules such as N-Acyl homoserine lactones (N.AHLs) in case of G-ve bacteria [49,129,130]. In particular, recent investigations reported that this pathovar produces N.AHLs which regulates the virulence and other biological activities [29,124].

**Figure 6:**
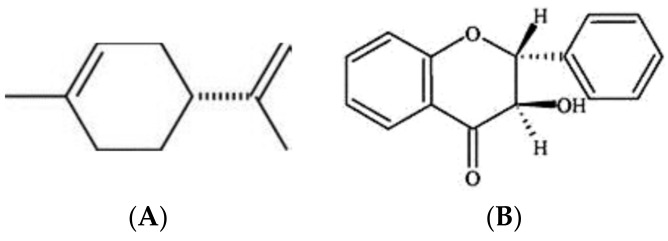

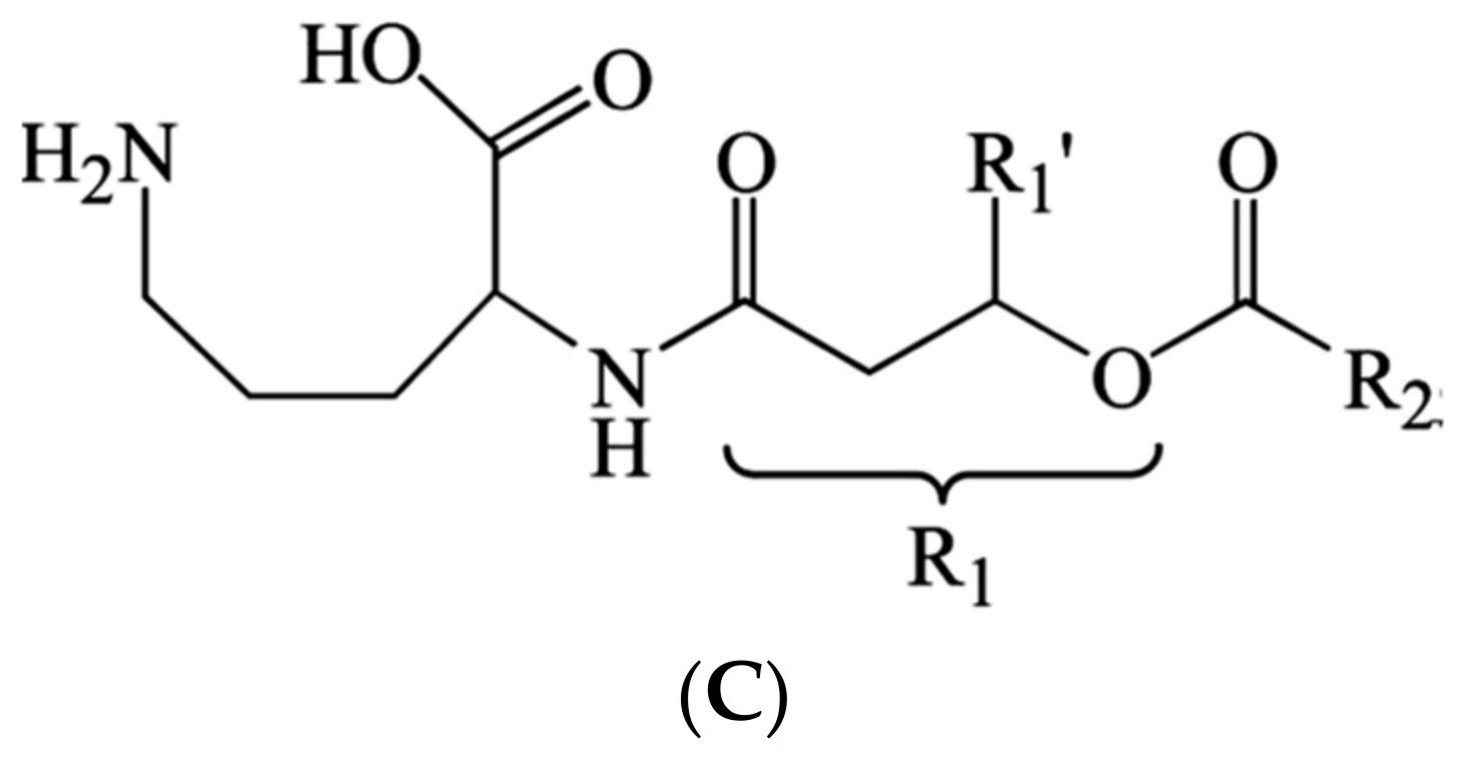
Secondary metabolites produced by *B. gladioli* pv. *agaricicola*. Where (**A**) D-Limonene, (**B**) 4-Flavanone, (**C**) Ornithine lipid.

Regarding the volatile organic compounds (VOCs) produced by *Bga*, they induced a reduction of fungal growth of *Fusarium oxysporum* and *Rhizoctonia solani* [131]. The biochemical characterization of VOCs produced by strain ICMP 11096 from this pathovar has identified two bioactive compounds. The first one was a liquid hydrocarbon cyclic terpene identified as cyclohexene 1-methyl-4-(1-methylethenyl) (Figure 6A), as the more frequent d-isomers of limonene [131]. The second one was identified as 4-flavanone (4H-1-Benzopyran-4-one, 2, 3-dihydro-2-phenyl) (Figure 6B). The two produced VOCs could be mainly responsible for the antifungal activity of this pathovar against phytopathogenic and plant-associated fungi [131,132].

The chemical analysis of the main diffusible secondary metabolites of *Bga* by using Liquid Chromatography-Mass Spectroscopy (LC-MS) and Nuclear Magnetic Resonance (NMR) investigations demonstrated that the main isolated bioactive diffusible substance is an amino lipid compound identified as ornithine lipid (Figure 6C) [31]. On the other hand, the same authors reported that the ornithine lipid represented a major polar lipid constituent of the whole bacterial cell.

In Table 2, we reported the most important secondary metabolites produced by the above seven species of *Burkholderia* spp. with their related references.

## 7. Conclusions

Genus *Burkholderia* is one of the most important group of plant, animal, and human associated bacteria. It is well-known for its virulence, bioactivity and microbicide properties. This genus includes different species which occupy wide range of ecological niches, such as *B. cepacia*, *B. andropogonis*, *B. plantarii*, *B. caryophylli*, *B. glumae* and *B. gladioli,* which are the causal agents for different plant, animal and human diseases. The current review deals with some important species of *Burkholderia* which have been manipulated in different studies. It is worth noting to underline that the study of metabolic profile of this genus could aid in revealing different aspects of this group related to its pathogenicity, virulence, plant-microbe interaction and role of produced metabolites in controlling phytopathogens. It is beyond doubt that the knowledge of synthesized secondary metabolites of this group will also support differentiation between different species and eventually strains and pathovars. Detailed information has been reported here regarding some important identified secondary metabolites from different species and pathovars of *Burkholderia*, their chemical structures, biological activities and modes of action against several phytopathogens. It is concluded that genus *Burkholderia* has important biological and metabolic properties and can be exploited in promising ways as antagonising biocontrol agents, for soil bioremediation and plant growth promoting purposes. Finally, different synthesized metabolites by *Burkholderia* can be used effectively in human and agro-pharmaceutical industry.

## Figures and Tables

**Table 1:** List of species of *Burkholderia* mentioned in the current review.

Species of *Burkholderia*		Author	Year of Description	Disease	Host
1	*B. alpina*	Weber and King	2017	-	-
2	*B. ambifaria*	Coenye et al.	2001	belongs to B. cepacia complex	
3	*B. andropogonis*	Smith (Gillis et al.)	1911, 1995	bacterial leaf stripe	sorghum and corn
4	*B. anthina*	Vandamme et al.	2002	belongs to *B. cepacia* complex	
5	*B. arboris*	Vanlaere et al.	2008	belongs to *B. cepacia* complex	
6	*B. caryophylli*	Burkholder (Yabuuchi et al.)	1942, 1993	wilt, stem cracking and rot of stems and roots	carnation
7	*B. cenocepacia*	Vandamme et al.	2003	cystic fibrosis	humans
8	*B. cepacia*	Palleroni and Holmes (Yabuuchi et al.)	1981, 1993	cystic fibrosis soft-rotting	humans onion
9	*B. contaminans*	Vanlaere et al.	2009	belongs to *B. cepacia* complex	
10	*B. diffusa*	Vanlaere et al.	2008	belongs to *B. cepacia* complex	
11	*B. dolosa*	Vermis et al.	2004	belongs to *B. cepacia* complex	
12	*B. gladioli*	Severini (Yabuuchi et al.)	1931, 1993	a. Scabdisease b. severe pulmonary infections c. soft rot	- gladiolus corms - humans - mushroom
13	*B. glumae*	Kurita and Tabei (Urakami et al.)	1967, 1994	panicle blight	rice
14	*B. humptydooensis*	Vanlaere et al.	2009	melioidosis disease	humans and animals
15	*B. lata*	Vanlaere et al.	2009	belongs to *B. cepacia* complex	
16	*B. latens*	Vanlaere et al.	2008	belongs to *B. cepacia* complex	
17	*B. mallei*	Zopf (Yabuuchi et al.)	1885, 1993	glanders disease	animals
18	*B. metallica*	Vanlaere et al.	2008	belongs to *B. cepacia* complex	
19	*B. multivorans*	Vandamme et al.	1997	belongs to *B. cepacia* complex	
20	*B. oklahomensis*	Glass et al.	2006	melioidosis	humans
21	*B. plantarii*	Azegami et al. (Urakami et al.)	1987, 1994	seedling blight	rice
22	*B. pseudomallei*	Whitmore (Yabuuchi et al.)	1913, 1993	melioidosis disease	humans and animals
23	*B. pseudomultivorans*	Peeters et al.	2014	belongs to *B. cepacia* complex	
24	*B. puraquae*	Martina et al.	2018	belongs to *B. cepacia* complex	
25	*B. pyrrocinia*	Imanaka et al. (Vandamme et al.), (Storms et al.)	1965, 1997, 2004	cystic fibrosis	humans
26	*B. rhizoxinica*	Partida-Martinez et al.	2007	rice seedling blight, associated with *Rhizopus microsporus*	rice
27	*B. seminalis*	Vanlaere et al.	2008	belongs to *B. cepacia* complex	
28	*B. singaporensis*	Wang et al.	2003	-	-
29	*B. singularis*	Vandamme et al.	2017	respiratory system disease	humans
30	*B. stabilis*	Vandamme et al.	2000	belongs to B. cepacia complex	
31	*B. stagnalis*	De Smet et al.	2015	*B. stagnalis*	
32	*B. territorii*	De Smet et al.	2015	belongs to *B. cepacia* complex	
33	*B. thailandensis*	Brett et al.	1998	melioidosis disease	humans and animals
34	*B. ubonensis*	Yabuuchi et al.	2000	-	-
35	*B. vietnamiensis*	Gillis et al.	1995	cystic fibrosis	humans

**Table 2:** List of secondary metabolites synthesized by some species of *Burkholderia* spp.

No.	Species	Synthesized Metabolites	References
1	*B. cepacia*	Pyrrolnitrin	[26,72]
		Cepacidine A	[75,76]
2	*B. andropogonis*	Rhizobitoxine	[81,82]
3	*B. plantarii*	Tropolone	[87]
4	*B. plantarii* strain DSM 9509	Rhamnolipids	[88]
5	*B. rhizoxinica*	Rhizoxin	[89,90]
6	*B. glumae*	Toxoflavin	[97,98]
7	*B. caryophylli*	Caryoynencine	[99,101]
8	*B. gladioli* pv. *cocovenenas*	Bongkrekic acid and toxoflavin	[108,116]
9	*B. gladioli* pv. *agaricicola*	d-Limonene	[127]
		4-Flavanone	[128]
		Ornithine lipid	[32]
